# Breaking the Circle at the Front Door: Violence, Burnout, and the Exodus From European Emergency Departments

**DOI:** 10.7759/cureus.111664

**Published:** 2026-06-28

**Authors:** Giuseppe Fumai, Ariangela Poliseno, Adriana Saracino, Gianluca Laricchia, Giuseppe Gisario

**Affiliations:** 1 Orthopedic and Neurological Rehabilitation, Rehcura Rehabilitation Facility, Adelfia, ITA; 2 Emergency Department, Azienda Sanitaria Locale (ASL) di Bari, Bari, ITA; 3 Emergency Department, Azienda Ospedaliero-Universitaria Consorziale Policlinico di Bari, Bari, ITA; 4 Nursing, Università degli Studi di Foggia, Foggia, ITA; 5 Nursing, Azienda Sanitaria Locale (ASL) di Foggia, Cerignola, ITA

**Keywords:** burnout, emergency department, emergency nursing, europe, intention to leave, organizational well-being, staffing adequacy, triage competence, workforce turnover, workplace violence

## Abstract

European emergency departments act as the pressure valve of their health systems, absorbing the demand that primary care, community services, and hospital capacity cannot. In this role they face persistent crowding, workplace violence, staff burnout, and difficulty retaining experienced professionals. We argue that these problems are not independent. They form a self-reinforcing organizational circuit, in which crowding strains triage, strained triage and prolonged waiting fuel violence, violence and understaffing fuel burnout, and burnout drives experienced professionals to leave. Turnover then degrades triage competence and increases exposure to violence, closing the loop. Fragmented, single-issue responses cannot interrupt a circuit of this kind. This editorial proposes a heuristic organizational framework, presented as a practice-oriented table rather than a validated standard. It integrates five domains: triage and process competence; staffing adequacy and workforce stability; security infrastructure and violence prevention, including fixed police presence at high-volume emergency departments; psychological support and well-being; and the community and territorial care interface, including population education on appropriate emergency department access. The framework is advanced as a normative, testable proposition to guide policy, local protocol development, and future empirical evaluation.

## Editorial

European emergency departments act as the pressure valve of their health systems. They absorb whatever primary care, community services, and hospital capacity cannot. Crowding is the visible expression of that role, and its consequences are no longer in dispute. A systematic review of 102 studies established poorer patient outcomes, and a reduced ability of staff to follow guideline-recommended treatment, as recurring effects of emergency department crowding [1]. The same review identified a structural mismatch at the heart of the problem. Most documented causes relate to the number and complexity of people attending and to delayed outflow from the department, whereas most evaluated solutions focus on internal patient flow. Whole-of-system initiatives and extended availability of primary care emerged as the more promising directions [1]. Crowding is therefore largely manufactured outside the emergency department, by weak territorial networks that neither filter inappropriate demand upstream nor accept patients downstream, yet its costs are paid inside, by patients and professionals.

Triage is where those costs concentrate first. It is the gatekeeping process through which clinical risk is stratified, waiting is legitimized, and the implicit contract between citizens and the emergency system is negotiated at the front door. No triage scale, however carefully validated, produces triage competence by itself. Competence lives in the trained professional who applies the scale under real conditions, such as crowded waiting rooms, incomplete information, and prolonged boarding that requires structured re-triage. It is precisely under these conditions that errors of under-triage, and the perception of arbitrary waiting, tend to arise.

That perception has a well-known sequel, which is violence. In the 2016 Italian National Survey of emergency nurses, 76.0% of respondents reported verbal violence and a further 15.5% reported both verbal and physical violence, leaving only 8.5% who had experienced neither. Older age and greater experience in emergency settings emerged as protective factors, whereas working in southern Italy significantly increased the probability of exposure [2]. The same study indicated that effective responses are necessarily composite. They include specific training for younger nurses, the rebuilding of an alliance of trust between users and health personnel, and physical barriers and appropriate architectural measures [2].

What is striking, against this epidemiology, is how thin the security infrastructure of many European emergency departments remains. Law-enforcement presence is often occasional, outsourced, or activated only after an event, so that the institutional response to violence is reactive by design. Where fixed police posts at high-volume emergency departments are absent, the most elementary form of deterrence is missing from the one hospital area where aggression is most predictable. Deterrence alone is not a strategy, and a punitive climate would be counterproductive. Yet visible and stable security presence, protective design of triage areas, and user-friendly reporting systems embedded in a just culture are complementary, not alternative, to the relational and educational measures that the evidence already supports [2].

Italy offers the most instructive European example of the legal route. Its epidemiology of emergency department violence is among the best documented in Europe [2]. Its legislative response is also among the most advanced. Law No. 113 of 14 August 2020 increased the criminal penalties for serious injuries inflicted on health and social care professionals, introduced a specific aggravating circumstance for violent offences committed against them, and established an administrative sanction for violent or abusive conduct toward them [3]. The same law required healthcare organizations to embed operational protocols with police forces in their security plans, and it established a National Observatory on the safety of health and social care professionals [3]. Yet the national survey data largely predate this legal architecture [2], and fixed police presence remains unevenly implemented across facilities. Italy is therefore a natural laboratory for the question this framework poses, namely whether penal deterrence, without parallel organizational investment, changes the daily exposure of triage nurses at all.

Violence is only one of the loads that emergency professionals carry. In a European Society for Emergency Medicine survey of 1,925 emergency professionals, conducted after two years of the COVID-19 pandemic, 62% met criteria for burnout. The proportion was higher among nurses than physicians (73% versus 60%), and higher still among professionals with less than five years of experience (74%) [4]. Respondents who reported frequent understaffing had markedly higher burnout than those who did not (70% versus 37%). Burned-out professionals reported the desire to change workplace far more often (87% versus 40%), and only 41% reported access to support programs [4]. The gap between the scale of the problem and the availability of structured psychological support is itself a policy finding. European emergency systems currently treat as optional what their own workforce data identify as essential.

The endpoint of this trajectory is attrition. In the RN4CAST study of 23,159 nurses across 10 European countries, 9% intended to leave the profession, with national figures ranging from 5% to 17%. Burnout was the factor most consistently associated with that intention, roughly doubling its odds, whereas perceived staffing adequacy and patient-to-nurse ratios were not directly associated at the European level [5]. This does not absolve staffing. It suggests instead that staffing inadequacy operates indirectly, through the exhaustion it produces [4,5]. Turnover is not merely a human-resources statistic. Because experience protects against exposure to violence [2], and because it is the substrate of reliable triage, every experienced professional who leaves makes the department both more dangerous and less competent, feeding the very conditions that drove the departure.

The picture that emerges is circular. Crowding generated upstream strains triage. Strained triage and prolonged waiting feed violence. Violence and understaffing feed burnout. Burnout feeds the intention to leave, and turnover then degrades triage competence and increases exposure to violence. Fragmented, single-issue responses, such as a surveillance camera in one place and a communication course in another, cannot interrupt a circuit of this kind. What is needed is an integrated organizational framework that acts at the same time on competence, staffing, security, well-being, and the community interface.

In light of these gaps, we propose a heuristic organizational framework for safety, retention, and well-being in European emergency departments. It should be read as a practice-oriented, testable model grounded in the preceding analysis, not as an empirically validated standard. Table 1 outlines how its five domains could translate into expected actions and operational strategies. The model is intentionally generic with respect to national health system architectures, so that it can be adapted to different organizational and legal contexts. It is deliberately framed in action-oriented terms, to promote alignment between hospital-level measures, law-enforcement cooperation, and territorial health policies.

**Table 1:** Heuristic organizational framework for safety, retention, and well-being in European emergency departments (proposed model) Authors’ own elaboration. The model is heuristic; its elements require local adaptation, piloting, and rigorous evaluation before any incorporation into policy, contractual, or accreditation frameworks.

Domain	Expected actions and competencies	Suggested operational strategies
Triage and process competence	Maintain certified, up-to-date triage competence; apply structured re-triage during prolonged waits and boarding; recognize and escalate clinical deterioration in waiting areas; monitor under-triage and over-triage as process indicators.	Structured triage education with simulation and case-based assessment; periodic competence revalidation; supervised induction and mentoring for nurses newly assigned to triage; routine audit of triage performance indicators with feedback loops.
Staffing adequacy and workforce stability	Match staffing to patient acuity and flow rather than fixed historical rosters; limit reliance on involuntary overtime; monitor turnover, vacancy rates, and skill-mix dilution as organizational safety signals.	Acuity- and flow-based staffing models; retention incentives and career pathways for experienced emergency staff; structured onboarding and succession planning; turnover and vacancy dashboards reviewed periodically by management.
Security infrastructure and violence prevention	Recognize early warning signs of aggression; apply de-escalation techniques; report all violent episodes, including verbal abuse; cooperate with security and law enforcement personnel within defined protocols.	Fixed police posts or stable law-enforcement presence at high-volume emergency departments; formal agreements with local police for rapid response; controlled access, video surveillance, and protective design of triage areas; user-friendly reporting systems within a just culture; zero-tolerance institutional policies; de-escalation training for all front-line staff.
Psychological support and well-being	Recognize early signs of burnout, moral distress, and post-traumatic reactions in oneself and colleagues; normalize help-seeking; participate in debriefing after critical or violent events.	Structured, easily accessible psychological support services for emergency staff; routine post-event debriefing; periodic burnout monitoring with validated instruments; peer-support programs; rota design that protects recovery time.
Community and territorial care interface	Inform and orient citizens toward appropriate emergency department use; redirect low-acuity presentations to alternative pathways where clinically safe; collaborate with primary and community care services on admission avoidance and discharge.	Population education campaigns on appropriate emergency department access; strengthening of territorial care networks (out-of-hours primary care, community hospitals, intermediate care); structured diversion and fast-track pathways for low-acuity patients; discharge coordination protocols with territorial services.

This table is intended as a heuristic tool to stimulate dialogue between hospitals, regional health authorities, law-enforcement agencies, universities, professional associations, and citizens’ representatives. It is not a prescriptive standard. Its elements require local adaptation, piloting, and rigorous evaluation before any incorporation into formal policies or accreditation frameworks.

For practice, adopting the framework would mean three things. Triage competence would be treated as a certified and periodically revalidated function rather than an assignment. Turnover, vacancy rates, and violence reports would be read as organizational safety signals, reviewed by management with the same discipline applied to clinical indicators. Emergency staff would be guaranteed protected access to psychological support and post-event debriefing as a structural service, not an emergency measure.

For policy, the model implies commitments that exceed the perimeter of any single hospital. The first is stable law-enforcement presence at high-volume emergency departments, negotiated at regional or national level. The second is investment in territorial care networks, including out-of-hours primary care, community hospitals, and intermediate care, capable of filtering demand upstream and accepting patients downstream. Strengthening primary care and managing demand upstream are among the most promising responses to crowding [1]. The third is sustained population education on appropriate emergency department access, treated as a matter of civic health literacy rather than individual blame.

For research, the first question is the most basic. Whether any proposed framework, including this one, can actually be implemented should not be assumed but investigated. This calls for a deliberate sequence of secondary and primary research that maps the existing evidence, identifies organizational and contextual gaps, and tests candidate solutions against them. Evidence syntheses, such as scoping and systematic reviews, can establish what is known about how these organizational factors are jointly addressed across European systems. Primary studies, from cross-sectional surveys to mixed-methods implementation research, can then probe the gaps that those syntheses expose. Within such a program, the framework points to several testable hypotheses. Emergency departments that combine certified triage competence, acuity-based staffing, and visible, stable security presence may report lower rates of violence and lower intention to leave. The availability of structured psychological support may moderate the association between understaffing, burnout, and turnover [4,5]. Strengthening the territorial interface may measurably reduce low-acuity demand and crowding [1].

This circle cannot be broken at a single point, because each of its links sustains the next. The editorial therefore advances a normative proposal rather than a definitive solution. It is a heuristic organizational framework that treats the safety, retention, and well-being of emergency professionals as a single system property, one that no security camera, training course, or recruitment campaign can deliver alone. Its value will depend not on its elegance on paper, but on the willingness of institutions to translate it into practice and to subject it to rigorous empirical scrutiny.
